# Atrial cardiomyopathy in endurance athletes

**DOI:** 10.1038/s44325-024-00032-8

**Published:** 2024-11-21

**Authors:** L. W. Spencer, P. D’Ambrosio, M. Ohanian, S. J. Rowe, K. Janssens, G. Claessen, D. Fatkin, A. La Gerche

**Affiliations:** 1 St Vincent’s Institute, Fitzroy, Victoria Australia; 2 University of Melbourne, Parkville, Victoria Australia; 3 Department of Cardiology, The Royal Melbourne Hospital, Melbourne, Victoria Australia; 4 Victor Chang Cardiac Research Institute, Darlinghurst, New South Wales Australia; 5 Cardiology Department, St Vincent’s Hospital Melbourne, Fitzroy, Victoria Australia; 6 Exercise and Nutrition Research Program, Mary MacKillop Institute for Health Research, Australian Catholic University, Melbourne, Victoria Australia; 7 KU Leuven, Leuven, Belgium; 8 School of Clinical Medicine, Faculty of Medicine and Health, UNSW Sydney, Kensington, New South Wales Australia; 9 Cardiology Department, St Vincent’s Hospital, Darlinghurst, New South Wales Australia

**Keywords:** Interventional cardiology, Medical imaging, Cardiovascular diseases, Physiology, Cardiology, Diseases, Health care

## Abstract

Atrial cardiomyopathy is characterized by electrical and structural remodeling of the atria, which can predispose to arrhythmias and thromboembolic stroke. Changes in atrial size and function are frequently observed in athletes engaged in endurance sports, a phenomenon known as “athlete’s heart.” Common left atrial observations in athletes may include larger left atrial volumes but lower left atrioventricular volume ratios, mildly reduced left atrial strain, possible mild left atrial fibrosis, longer P-wave duration, and greater atrial ectopic activity. However, it remains unclear whether these changes represent physiological adaptations to endurance exercise or disease-promoting pathology. While the athlete’s heart is considered a benign physiological phenomenon, endurance athletes have an established risk of atrial fibrillation. Therefore, atrial cardiomyopathy represents a significant consideration in disease prognostication and the development of management strategies for athletes. This review examines current literature with respect to the clinical features, causes, and consequences of atrial cardiomyopathy in athletes.

## Introduction

**Fig. 1:**
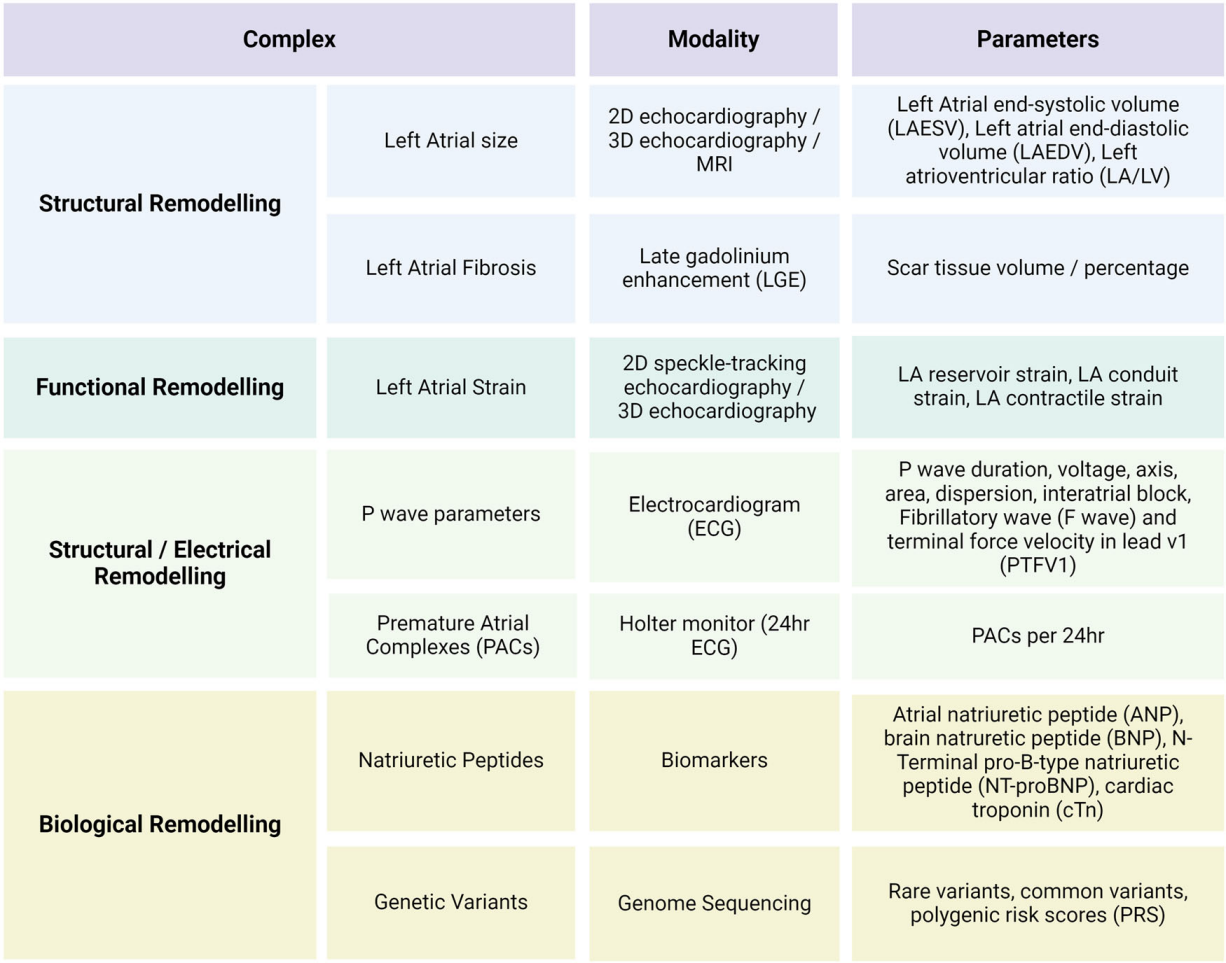
Atrial-specific physiological and functional considerations. Analysis of left atrial cardiomyopathy includes any complex of structural, functional or electrophysiological remodeling that may be clinically relevant. Created with BioRender.com.

**Fig. 2:**
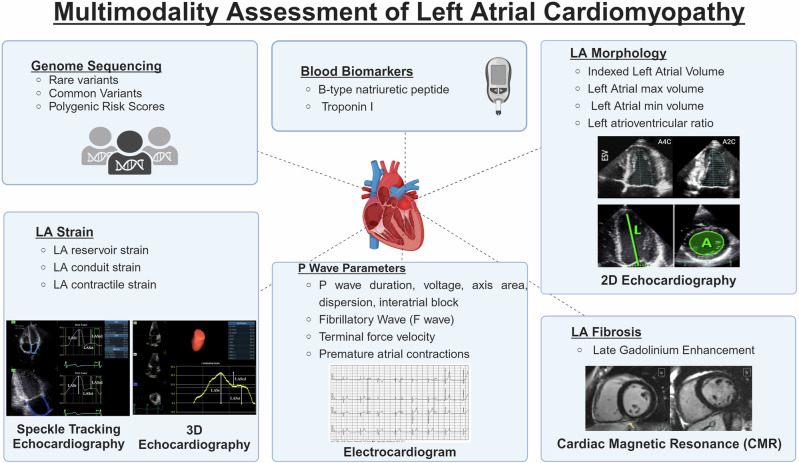
Multimodality assessment of left atrial cardiomyopathy. The assessment of left atrial cardiomyopathy necessitates the utilization of multiple assessment modalities, including cardiac magnetic resonance, electrocardiogram, echocardiography, genome sequencing and blood biomarkers. Created with BioRender.com.

Atrial cardiomyopathy (AtCM) has been defined as “any complex of structural, architectural, contractile or electrophysiological changes affecting the atria with the potential to produce clinically relevant manifestations” (Fig. 1)^1^. The etiology of AtCM is multifactorial, with age, genetic predisposition, and a range of cardiac and systemic disorders being identified as contributing factors. Pathophysiological mechanisms underpinning AtCM include inflammation, oxidative stress, atrial stretch and neurohormonal signals. Four classes of AtCM have been proposed, based on histological characteristics: (1) primarily cardiomyocyte changes (e.g., cell hypertrophy and Myocytolysis), (2) principally fibrotic changes with normal cardiomyocyte appearance, (3) combined cardiomyocyte and fibrotic changes, and (5) alteration of interstitial matrix^1^. The reliance of this classification method on atrial tissue analyses limits its practical application, and clinical evaluation of the presence and severity of AtCM is challenging. AtCM represents a discrete pathological entity that serves as an underlying substrate for the manifestations of atrial arrhythmia, atrial thrombogenesis and valvular dysfunction. Current approaches to the diagnosis of AtCM rely on multimodality assessment (Fig. 2). Due to its fluid definition, few studies have created clinically useful diagnostic criteria to define AtCM. The large ARCADIA trial defined AtCM as any electrical, biological or structural abnormality, as evidenced by the presence of either: P-wave terminal force in lead V1 (PTFV1) >0.05 mm·s, N-Terminal pro-B-type natriuretic peptide (NT-proBNP) >250 pg/mL or left atrial diameter >3 cm/m^2^ ^2^. The latest consensus update has proposed a categorization of AtCM as mild, moderate and severe^3^. Mild AtCM is characterized by subclinical remodeling without overt arrhythmia or significant mechanical dysfunction. Moderate AtCM is characterized by clinically significant structural abnormalities, functional abnormalities, elevated serological biomarkers (BNP/ANP, etc.) and manifestations of atrial fibrillation (AF). Severe AtCM is characterized by either systolic failure of atrial function (LA ejection fraction (EF) ≤35%), impaired atrial contractility (defined as flow velocities ≤20 cm/s within LAA/tissue strain), significant tissue alteration (atrial fibrosis, fatty infiltrates, amyloid infiltration, inflammation), severe LA enlargement (diameter ≥5.5 cm or LAVi ≥50 mL/m^2^) and/or longstanding persistent or permanent AF.

Endurance athletes represent a subpopulation of interest for AtCM as they undergo cardiac adaptations, but also have a well-established increased prevalence for an AtCM endpoint of AF. This review outlines methods for assessing AtCM, with a particular focus on contextualization for endurance athletes. Exercise-induced atrial remodeling can be extreme in this context, but whether this represents a physiological or pathological response is uncertain. This is a clinically important issue with implications for prognosis, participation in sports and the formulation of exercise recommendations.

## LA structural markers of atrial cardiomyopathy

In the general population, left atrial (LA) size has been established as a marker of cardiovascular disease due to its strong association with AF, stroke and mortality^4–6^. Measures of LA structure and function have proven prognostic value in the assessment of left ventricular (LV) diastolic dysfunction (LVDD) and heart failure^7^. LA structural changes include chamber dilation, cardiomyocyte hypertrophy, fibrosis, infiltration of fat and deposition of amyloid fibrils. LA dilation, often referred to as LA enlargement (LAE), is defined with respect to population reference ranges and is typically assessed by transthoracic echocardiography (TTE). The American Society of Echocardiography and European Association of Cardiovascular Imaging reference values are the same, irrespective of biological sex, and define the normal range for maximal LA volume indexed to body surface area at end-systole (LAVi), as 16–34 mL/m^2^, with severity partition cutoffs of 35–41 mL/m^2^ as mildly abnormal, 42–48 mL/m^2^ as moderately abnormal and >48 mL/m^2^ as severely abnormal^8^. In clinical practice, 2-dimensional TTE is widely utilized to evaluate LA size, but 3-dimensional TTE and cardiac magnetic resonance (CMR) have potential advantages. CMR can be used to measure atrial volumes, although careful planning to maximize long-axis dimensions is required to avoid ‘foreshortening’ that can lead to marked underestimation of volumes.

Fibrosis plays a pivotal role in the development of AtCM^9–11^. Quantification of LA fibrosis using late-gadolinium enhancement (LGE) has been reported in some expert centers, but the reproducibility of this promising technique from the Comprehensive Arrhythmia Research & Management Center (CARMA) is yet to be established^12^. Electro-anatomical voltage mapping using multipolar electrode catheters can also be utilized to identify areas of low voltage that could indicate fibrosis. Patients with AF have been found to have lower LA voltage compared to controls^13^.

Contextualizing LAE and identifying individuals with disproportionate left-sided remodeling could improve understanding of pathology. LA and LV size and function are interdependent, as the LA serves its role as a priming pump by filling the LV. N-LA/LV volume ratios could be used to differentiate potential AtCM, especially in the absence of strain. Disproportionate LA/LV volume ratio has been associated with outcomes in a large cohort of individuals with subclinical cardiovascular disease^14^. Similarly, in healthy subjects, the LA/LV volume ratio has utility in the assessment of LVDD as it is associated with diastolic markers, including LV filling pressures (E/e’) and pulmonary artery pressure^15^ (10.1093/ehjimp/qyae028). These findings suggest that an increased LA/LV volume ratio is indicative of disproportionate remodeling and could be a marker for AtCM risk.

## LA function and atrial cardiomyopathy

LA speckle tracking echocardiography can be used to measure atrial strain and has emerged as a valuable and reliable tool for assessing LVDD and atrial function^16–19^. The LA functions as a reservoir for pulmonary venous return during end-systole, a conduit for pulmonary venous return during early ventricular diastole and a contractile/booster pump that increases ventricular filling during late diastole^20^. LA strain has clinical utility as it reflects the pressure-volume relationship and essentially incorporates several parameters into a singular measurement. In an at-risk population, LA reservoir strain has been demonstrated to correlate with age, e’, LAVi and LV global longitudinal strain^21^. LA strain has garnered interest as a prognostic marker in LVDD assessment as mechanical LA function is more sensitive than structural measurements of LA size and therefore abnormalities in deformation occur earlier into the maladaptive remodeling process^22,23^. LA strain may also independently correlate with the extent of fibrosis^24^.

Considering the interdependence of the LA and LV, it is unsurprising that the LA/LV volume ratio and strain loops are strongly correlated^25^. Interestingly, the relationship between LA volume and LA strain may actually be non-linear, as the upper tertiles of LAVi have been found to have an exponential effect on LA reservoir strain^21^. In pathological cohorts of subjects with heart disease, there appears to be a positive correlation between LA strain parameters and exercise capacity, with higher strain values associated with greater cardiorespiratory fitness (CRF). In pathological AF and heart failure cohorts, impaired LA reservoir strain is associated with reduced exercise tolerance^26,27^. Furthermore, in patients with hypertrophic cardiomyopathy, exercise intolerance, defined as a peak oxygen uptake less than 80% of predicted value, was associated with lower reservoir, conduit, and contractile strain^28^.

Similar relationships between LA strain and CRF have been observed in healthy control cohorts. Following two years of exercise training in a randomized control trial of healthy sedentary participants, those prescribed exercise demonstrated increased LA chamber volumes and improved LAEF and no changes in pulmonary capillary wedge pressure, a surrogate of LA pressure^29^. Individuals prescribed exercise presented with larger LV volumes and a shift in the pressure-volume curve, indicating reduced stiffness and improved chamber compliance^29,30^. The impact of exercise training in the general population is not thought to be associated with reductions in LA strain, as LA strain is inversely related to LA pressure overload^31,32^. LA changes throughout exercise are unclear, but in healthy controls, LA reservoir volume index has been shown to remain unchanged throughout exercise due to decreased LA volumes at end-systole and end-diastole but increased LAEF^33^.

## ECG parameters in atrial cardiomyopathy

Changes in calcium cycling, ion channels and gap junctions can lead to electrophysiological changes in the LA^34^. The passage of electrical impulses through the atria is reflected in the electrocardiogram (ECG) as the P-wave^35^. The P-wave is composed of two overlapping components, the first phase dominated by right atrial contraction and the latter phases by the LA. ECG parameters relating to atrial innervation and function include P-wave duration, voltage, axis, dispersion, inter-atrial block, Fibrillatory wave (F) and PTFV1, with the latter reflecting both the duration and amplitude of atrial electrical activity. Marked LAE can be associated with a broad, notched (bifid) P-wave in lead II (P *mitrale*) and an accentuation of PTFV1. Ion channel remodeling affects action potentials and voltages in the LA. Impaired inter-atrial conduction and P-wave prolongation are both ECG findings characterized by P-wave duration but represent different aspects of atrial conduction^36^. Normal ranges for P-wave morphology include a duration ≤120 ms, voltage ≤0.25 mV and PTFV1 ≤0.04 mm·s^37^. Current normal ECG reference values also recommend adjustment for age and sex^38,39^. A more recent study analyzing the prevalence of altered PTFV1 in the general population revealed that 4.8% of individuals demonstrated a PTFV1 between 0.04–0.049, 1.5% between 0.05 to 0.059 and 1.2% ≥0.06 mm·s^40^. In the general population, PTFV1 has been shown to have a dose-dependent association with incident AF and stroke^41^. However, in comparing the influence of amplitude and duration on PTFV1, P-wave duration has a dose-response relationship with outcomes, whereas the relationship between amplitude and AF is less apparent^41^. P-wave dispersion, defined as the difference between the longest and shortest P-wave duration from ECG, is a useful tool for assessing the homogeneity of atrial electrical activity and could also be a promising method for predicting the recurrence of paroxysmal AF^42,43^. Premature atrial complexes (PACs) or short runs of PACs are also thought to play a role in AtCM. PAC activity has been proposed as an independent predictor of AF. In a population cohort of the Copenhagen Holter Study comprising 678 healthy participants aged 55–75 years with no history of cardiovascular disease, those with >30 PACs per hour or any single run of ≥20 PACs over a 48-h ambulatory ECG exhibited an increased incidence of AF, stroke and death, over a 6-year follow-up period^44^. PACs were also found to be associated with incident AF in the Cardiovascular Health Study, which observed 1260 adults without prevalent AF using 24-h ECG^45^. Although the prevalence of PAC is low, overlap of markers is rare. In a healthy population, an analysis of the overlap of potential AtCM markers using ECG, 24-h ECG and echocardiogram revealed that 7% of the population had more than 500 PACs, 19% had a P-wave duration >120 ms and 15% had LAVi ≥34 mL/m^2^, while only 9% presented with 2 markers and 0.3% had all 3 markers^46^.

## Other markers in atrial cardiomyopathy

Cardiac biomarkers are widely used to assess and monitor cardiac pathology. Biochemical changes in secretion that serve as indicators of cardiac stress include NT-proBNP, which reflects myocardial wall stress and cardiac troponin (cTn), which is a sensitive marker of myocardial damage. BNP is useful in the assessment of AtCM as it has added value when added to AF risk prediction models^47^. Although the role of cTn in AtCM is less clear, cTn may be useful in the assessment of AtCM, as cTn elevation is associated with cardiovascular comorbidities and predicts major adverse events in patients with no clear diagnosis^48^.

## Atrial cardiomyopathy in endurance athletes

AtCM could be pertinent to endurance athletes, given the observed atrial remodeling and increased prevalence of AF. The pressure and volumetric demands of frequent endurance exercise lead to profound cardiac remodeling, termed the athlete’s heart^49^. Under the broad definition of AtCM, the athlete’s heart presents as a clinical conundrum, as the accumulated exposure to transient volume overload from exercise leads to cardiac remodeling that can resemble pathology. Concern that exercise-induced atrial remodeling may not be benign is raised by the repeated observation that endurance athletes have a greater prevalence of AF than age-matched non-athletes^50–53^. In a study of 52,755 long-distance skiers, 919 individuals experienced arrhythmias, with those who participated in the greatest number of races demonstrating the highest incidence of AF^53^. Contextualization plays an integral role in the interpretation of the athlete’s heart. Factors including age, biological sex, ethnicity, genetics, and clinical risk factors all may contribute to atrial remodeling. Importantly, no athlete is the same. Significant inter-individual variations in cardiac remodeling occur between athletes due to differences in sporting code, level of competition and training load (intensity and duration)^54^. Differentiating between physiological and pathological remodeling may have important clinical implications^55^.

## Structural changes in athletes’ atria

**Fig. 3:**
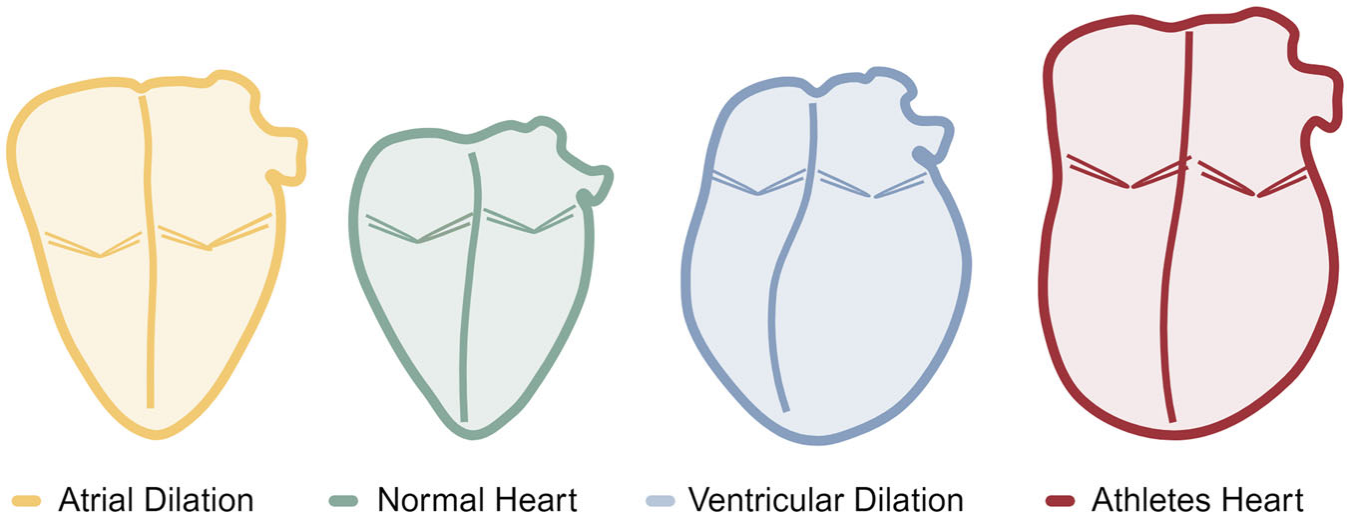
Proposed structural overlap in four categories of patients. Yellow = atrial dilation, green = normal heart, blue = ventricular dilation, red = athletes’ heart (bi-atrial and bi-ventricular dilation). Figure reproduced from Malaescu et al. with permission from Elsevier^25^. The figure was created using BioRender.com.

The athlete’s heart is associated with bi-atrial and bi-ventricular remodeling that can resemble disease pathologies, such as genetic cardiomyopathy (Fig. 3). Although the LV is most closely linked to CRF, upstream factors are also involved in the remodeling process, as there is a demonstrated positive relationship between CRF and LA volume^56,57^. LA volumes have been demonstrated to be at least 30% greater in athletes than non-athletes^58^. LA volume has a positive correlation with CRF, and therefore, it is to be expected that the fittest athletes will present with greatest physiological LA remodeling. This rationale extends to sporting codes, as athletes in sports requiring the highest CRF (cross-country skiing, cycling, triathlon, rowing) will have greater cardiac chamber dilation to sustain high cardiac output during exercise. The positive relationship between maximal oxygen consumption and LA chamber enlargement during exercise suggests that in endurance athletes, a degree of LA dilation should be expected.

Although fibrotic tissue is an important marker for the atria of athletes, the question of whether the atria of athletes are affected by fibrosis remains unanswered. A number of studies have sought to determine whether intense exercise induces inflammation and fibrosis of the atria. Results from animal models suggest that 16 weeks of exercise training, equivalent to 10 years of human exercise, promotes an inflammatory atrial response including atrial dilatation, enhanced vagal tone and atrial fibrosis. However, the authors note that the 30–60% increase in fibrous tissue was relatively limited compared to other animal models of atrial pathology^59^. Furthermore, LGE findings of LA fibrosis from the CARMA research center have also been observed in athletes. This intriguing observation, made in a relatively small cohort of athletes, requires validation in larger athletic populations and at other research centers^60^. Interestingly, interprocedural bipolar voltage mapping of 16 endurance athletes undergoing AF ablation indicates a low incidence of LA scarring, with only one athlete (6.3%) exhibiting LA scarring on bipolar voltage mapping^61^. Notably, the LAVi of these older athletes was normal (mean = 34.4 mL/m²), which is lower than what would be expected in a normal athletic LA, even in the absence of AF, where one would expect to observe further LA enlargement.

## Functional changes in athletes’ atria

The measurement of LA strain using echocardiography analyses mitral annulus displacement, along with volumetric changes in the LA and LV. Despite the clear relationship seen in athletes, where a bigger LA volume is associated with higher CRF, the relationship between CRF and LA function remains less clear. A number of studies have suggested that LA reservoir strain may be reduced in athletes^62,63^, whereas a positive correlation between CRF and LA reservoir strain observed by the authors would suggest that athletes have normal LA function^57^. The lack of consistency in the literature is largely due to LA strain parameters being weak correlates of CRF. A recent study published by our group showed that after taking age, sex, and LA volume into account in a population encompassing the CRF spectrum (including elite endurance athletes), LA strain parameters did not independently predict CRF^57^. Although LA function and LA volume are intrinsically linked, research has suggested that LA reservoir strain in athletes occurs independently of LA structural change^64,65^. In the general population, LA reservoir strain <18% and LA contractile strain <14% have clinical utility in identifying abnormal LV pressures^66^. In athletes, LA reservoir strain is seldom less than 18%, but LA contractile strain is quite commonly <14%^57^. As athletes typically present with normal or marginally reduced LA reservoir strain, the dilated LA chamber volumes in athletes are indicative of enhanced diastolic function. It has been demonstrated that endurance athletes may exhibit a lower LA contractile strain than non-athletes^57,62,63^. However, the clinical consequences of reduced LA contractile strain in athletes have not been established. Athletes with either LA reservoir strain <18% or LA contractile strain <14% should be interpreted with caution and may warrant further evaluation. With regard to physiology, the reduced LA contractile strain in athletes could be attributed to a shift toward earlier filling at rest, where parameters such as mitral E/A are elevated in athletes, suggesting high LA pressure^66^. Interestingly, conduit strain has a positive relationship with exercise, and therefore, the ratio of conduit/contractile LA strain may be a more robust predictor of exercise capacity.

It is important to recognize that echocardiography measurements are conducted at rest. In athletes, states of rest could reflect the phenomenon of a ‘hibernating heart’ where at rest, hemodynamic demands are minimal compared to maximal capacity. A number of studies have also assessed atrial changes post-exercise. Sanz-de la Garza et al. observed that atrial changes were more pronounced in the right atrium of 55 healthy adults following either small, medium or long trail running races. In the medium and long trail running races, LA strain parameters exhibited similar trends to changes in the RA, though they were not statistically significant^67^. Although LA strain measurements during exercise become challenging when E/A filling waves typically merge at heart rates above 100 bpm, future comparisons between athletic and non-athletic populations could prove useful in differentiating exercise responses. Athletes typically present with supernormal diastolic function and phenomenal filling rates compared to the general population. The enlarged volumes of athletes may also mean that lesser deformation is required to move similar stroke volumes. Consequently, the observed shift toward earlier filling during early diastole likely results in a reduced need for a final booster/kick LA contraction phase during late diastole. This reduced LA contractile strain in athletes may be restricted to young athletes since master athletes have relatively lower CRF and a demonstrated shift in filling toward late diastole^68^.

## ECG parameters in athletes’ atria

The 12-lead ECG is commonly used as a diagnostic and prognostic tool in the evaluation of the athlete’s heart due to its non-invasiveness, portability, and affordability. The ECG can be used to help discern normal structural and electrical alterations in athletes from abnormal findings requiring further investigation^69–71^. Based on the 2017 International ECG Recommendations for Athletes, LA dilatation and Left axis deviation observed on ECG should be regarded as a borderline variant not requiring further cardiologic assessment, unless accompanied by other abnormalities. However, these criteria are particularly relevant to sudden cardiac death, not AF^70^. The prevalence of ECG abnormalities varies significantly amongst athletic populations, with age and CRF levels representing significant confounding factors. In a study that compared 1000 junior athletes with a mean age of 16 to 300 non-athletic controls, it was found that 14% of the young athletes exhibited a terminal portion more negative than −0.1 mV and 0.04 s or more in duration (i.e., PTFV1 above 0.04 mm·s), compared to 1.2% in non-athletes^72^. P-wave duration and P-wave dispersion have been observed to be increased in young athletes^73^. However, the observed increase in dispersion is attributed to a significant decrease in the minimum P-wave duration. Findings from Herrera et al. comparing LA enlargement and ECG parameters in 308 athletes of skilled, strength resistance, or mixed activity, suggest that competitive training in athletes was not associated with longer P-wave duration or presence of IAB^74^. They did find that athletes with LA enlargement were more likely to have longer P-wave duration and a higher prevalence of inter-atrial block. Thus, it is plausible that in a cohort of endurance athletes where atrial dilation is common, P-wave duration is likely longer and elite endurance athletes may have a higher prevalence of IAB.

PACs have demonstrated linkage to AF occurrence and cardiovascular endpoints^75^. However, existing literature on atrial electrical signaling in athletes is inconclusive, and reference values are largely inconsistent. A study comparing lifetime exercise duration in non-elite athletes found that the highest exercise volume was associated with higher prevalence of P-wave prolongation, higher LA volume, higher vagal activity and more PACs^76^. However, another study found that PACs were not significantly higher in ex-professional cyclists^50^. ECG analysis on endurance athletes throughout exercise found that athletes had higher P-wave voltage post-exercise but an overall low prevalence of exercise-induced PABs^77^. Despite the conflicting evidence on PAC frequency in athletes, all studies found a low PAC compared to reference values, suggesting that >500 per 24 h indicates a high PAC burden^78^. The low prevalence of these athletic findings but clinical relevance of PAC to pathology suggests that frequent PAC activity may be a risk factor for arrhythmia and AtCM in athletes.

## Other changes in athletes’ atria

Acute exercise has been associated with elevated concentrations of both cTn and NT-proBNP^79,80^. A meta-analysis of 26 studies showed that post-exercise cTn levels exceeded the assay detection limit in almost half of the endurance athletes that had been studied^81^. However, resting NT-proBNP levels in endurance athletes are typically within normal reference values and have been observed to be similar in athletes and non-athletic subjects^82,83^.

## Overlap between physiological and pathological atrial remodeling

**Fig. 4:**
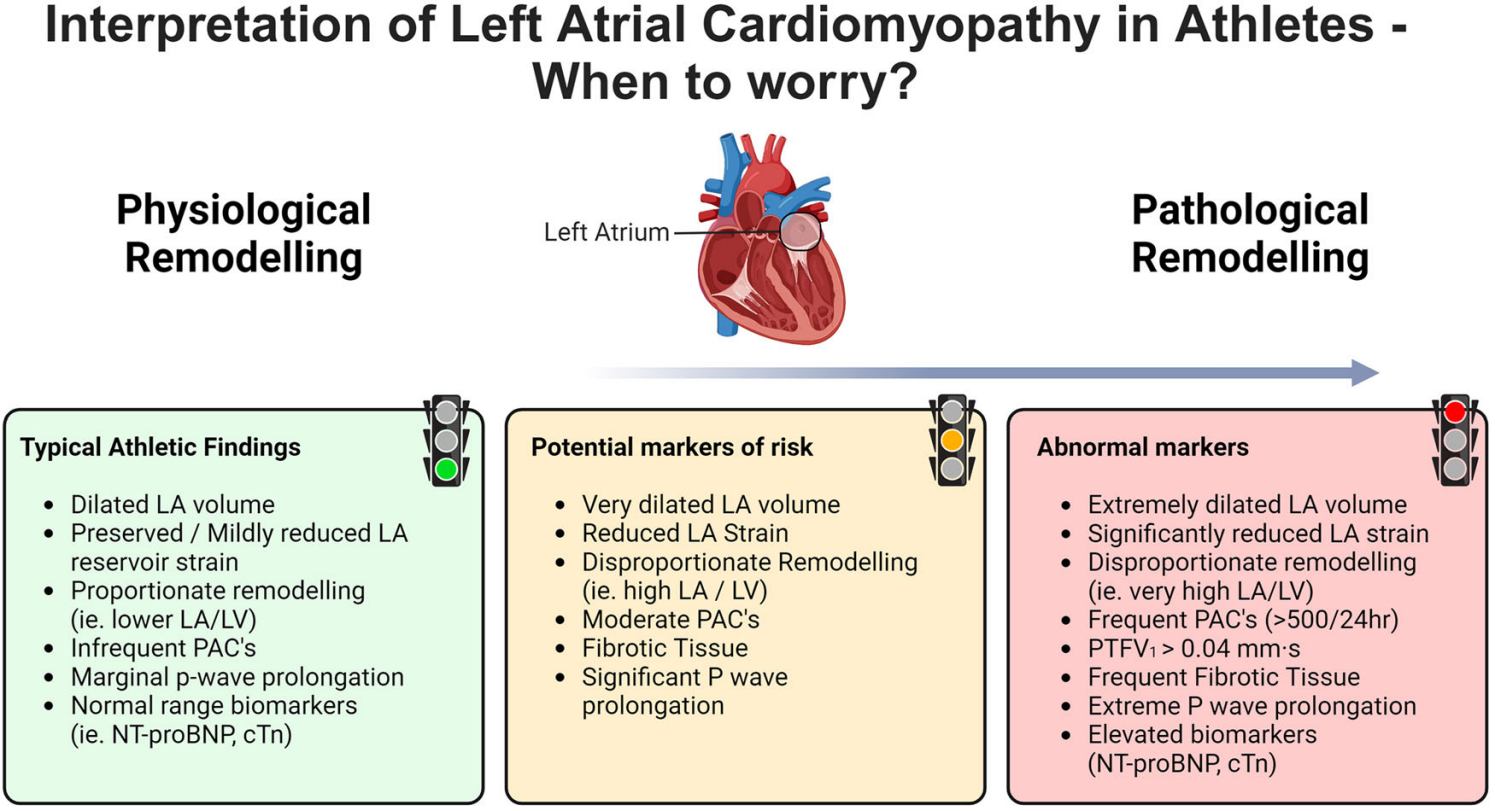
Interpretation of left atrial cardiomyopathy features in athletes. Green = normal athletic findings, amber = potential markers of risk, red = abnormal LA features that require further examination. Created with BioRender.com.

Exercise-induced atrial remodeling is generally considered a normal physiological response, however there are many features that overlap with pathological changes (Fig. 4). Although the athlete’s heart is regarded as a benign physiological phenomenon, endurance athletes are known to have an increased risk of complications of AtCM, such as AF^84^. Notwithstanding increased AF risk, athletes as a group have excellent health and have an increased lifespan compared to the general population^85^. A recent study of 144 participants compared 36 athletes and non-athletes, with and without AF^86^. The authors observed that AF was associated with LA dilation in non-athletes but that LA dilation was profound in athletes regardless of whether they had AF. Similarly, lower strain was associated with AF in non-athletes but was universally low in the athletes^86^. Therefore, the phenotypic overlap of LAE in athletes is unlikely to be a strong discriminator of AtCM. In both healthy and disease populations, the relationship between LA reservoir strain and risk appears to be inverse, with improvements in CRF associated with higher LA strain and improved clinical outcomes. However, the relationship between LA reservoir strain, specifically in athletes alone, appears to be less clear. Collectively, these findings further emphasize that the clinical significance of LA remodeling in athletes constitutes a “gray zone” where delineating boundaries between physiological and disease-promoting changes is challenging. This also raises a diagnostic conundrum for differentiating exercise-induced changes from those that might arise from coincident pathologies such as genetic cardiomyopathies.

Lifestyle risk factors and comorbidities influence AF risk in the general population. However, athletes do not typically present with the proarrhythmic risk factors that are typically associated with AF. Indeed, athletes typically exhibit a low prevalence of risk factors such as obesity, smoking, diabetes, sleep apnea, and hypertension^87,88^. Alcohol consumption does however represent as a potential AF risk factor that is prevalent in both athletes and the general population^89^. Although alcohol consumption is less frequent in elite athletes due to its negative impact on performance, alcohol consumption is a risk factor for AF that is often considered greater in athletes due to binge drinking culture^90^. Overall, athletes would score very low on AF lifestyle prediction scores that are informative in the general community^91,92^. This highlights the disparity between clinical AF risk scores and the heightened prevalence of AF in athletic cohorts.

## Defining risk in exercise-induced atrial cardiomyopathy

The mechanisms contributing to AF are likely different in athletes and the general population. Therefore, it begs the question as to when to worry and what markers are most useful in identifying the subset of athletes at greatest risk for AF. Importantly, any physiological LA remodeling should also be accompanied by even greater LV remodeling. The mechanisms that contribute to increased AF prevalence in endurance athletes still remain largely unclear. Potential factors contributing to AF include altered LA substrate, atrial ectopic activity and increased vagal tone^93^. LA reservoir strain in athletes typically falls within normal values, and therefore low values could be regarded as suspicious. However, LA strain is influenced by atrial size and function, and thus atrial dilation in athletes will contribute to marginally lower strain values^21^. PAC frequency in athletes is typically low in comparison to reference values and hence a high frequency of PACs in athletes could be considered abnormal^78^. The assessment of atrial structure and function must be made with an understanding of what constitutes the athletic norm after contextualization to age, sex, sport, training volume and level of competition. Common observations in athletes may include larger LA volumes but lower LA/LV volume ratios, slightly reduced LA strain, possible mild atrial fibrosis, longer P-wave duration, and greater atrial ectopic activity. Athletes with extreme atrial features that fall outside the athletic norm should be evaluated and followed closely for the presence of comorbidities.

## Sex differences in atrial cardiomyopathy in athletes

AtCM presents with sex-specific differences and issues that extend across the CRF spectra^94^. In athletes, distinctly abnormal LA remodeling is more prevalent in males than females^95^. However, when interpreting sexual dimorphisms, male athletes consistently demonstrate a greater exercise ceiling, which is typically accompanied by more profound cardiac remodeling, even after indexing to body size. Notably, reservoir strain appears to be lower in male athletes^57,63^. LA contractile strain was also found to be reduced in male athletes with moderate- and high-cardiac demand sports, whereas in females, contractile strain was not different based on cardiac demands^65^.

Overall, male athletes are at greater relative and absolute risk of AF when compared with female athletes. It was previously thought that only male athletes were at greater risk of AF, but recent literature suggests that female endurance athletes are also at a heightened risk for AF when compared to females of equivalent age in the general population^96^. Although the relative risk for females was lower than males, the cumulative prevalence of AF in female athletes was 15.6% (95% CI 6.4–29.4), compared to 10.3% (8.7–12.1) in female non-athletes^96^. In females, estrogens are thought to be cardioprotective, as they inhibit myocardial hypertrophy and proliferation of cardiac fibroblasts. There are age-related hormonal effects in females since AF risk increases significantly following menopause^97^.

## Genetics and atrial cardiomyopathy

Individual genetic variability likely contributes to the risk of AtCM development and AF, but data to support this are limited^98^. Genome-wide association studies (GWAS) have identified more than 100 genetic loci associated with AF^99^. Polygenic risk scores, derived from GWAS data, have recently been shown to have clinical utility beyond traditional markers in portending AF risk^100–102^. GWAS have also identified genetic determinants of echocardiographic traits^103^. Individual athletes might also carry rare genetic variants that influence AF risk or cardiomyopathic traits. A number of rare variants have been linked to early-onset AF, with mutations in *KCNQ1* and *TTN* genes being frequently identified as the causes of this condition^104–107^. In athletes with suspicious features such as extreme remodeling or frequent arrhythmias, genetic testing may be useful to exclude rare variants in genes that are associated with inherited cardiomyopathies. Whether there are gene-environment interactions is currently unexplored. It is plausible that exercise-induced atrial stretch may act as a “second hit” in individuals who have a genetic susceptibility to AtCM^108^. While genetics remains largely a research tool, athletes possessing single nucleotide variants that are predictive of risk should be monitored with caution throughout their athletic careers. Whether polygenic risk scores for AtCM or AF have prognostic utility in athletes has yet to be determined.

## Future directions

Future studies need to provide a deeper mechanistic understanding of AtCM in athletes and refine diagnostic criteria. Resting measurements tell only part of the story in the athletes’ atria, as LA strain would plausibly increase with exercise, and athletes may have an increased capacity for LA strain augmentation during exercise. Future analysis using CMR or stress echocardiography should assess LA strain response during exercise and determine whether athletic status is involved. Assessment of LA relies on multimodal imaging. Future studies on AtCM in athletes should assess the prevalence and overlap between potential risk markers. It is also possible that functional changes in the left atrium may be reflected in changes to the right atrium. This is an area of significant interest, given the observation that sinus node dysfunction may be more common in athletes^50^. Although AF is less common in female athletes, future research should try to identify whether sex-specific markers are involved in AF risk. The advent of artificial intelligence using deep learning convolutional neural networks has emerged as a significant advancement for in-depth ECG analysis, offering further insights that are largely unrecognizable to human interpretation^109,110^. This promising research technique has the potential to better predict AtCM endpoints of AF in the general population and could be extended to athletes. Similarly, deep learning networks could be extended to other imaging modalities, such as echocardiography and CMR, although this methodology depends on the availability of very large datasets that may not be available in athletic populations.

## Data Availability

No datasets were generated or analysed during the current study.
